# Editorial: Deciphering immunological mechanisms of spatial and temporal vaccine delivery

**DOI:** 10.3389/fimmu.2024.1354796

**Published:** 2024-02-27

**Authors:** Mingzhao Zhu, Anna Katrina Walduck, Wei Lu

**Affiliations:** ^1^ Key Laboratory of Infection and Immunity, Institute of Biophysics, Chinese Academy of Sciences, Beijing, China; ^2^ College of Life Sciences, University of the Chinese Academy of Sciences, Beijing, China; ^3^ Rural Health Research Institute, Charles Sturt University, Orange, NSW, Australia; ^4^ Key Laboratory of Smart Drug Delivery Ministry of Education, State Key Laboratory of Molecular Engineering of Polymers, School of Pharmacy, Fudan University, Shanghai, China

**Keywords:** vaccine, adjuvant, targeted delivery, nanovaccine, immunological mechanism

The use of vaccines to prevent infectious diseases represents a tremendous accomplishment of biomedical science and clinical practice. At a global level, life expectancy has increased from 46 years in 1950 to 73 years in 2023 (1), progress that has been aided by improvements in sanitation, healthcare and the introduction of vaccines against childhood diseases from the 1930s (2). However, there are still many unmet needs, and questions surrounding vaccine design. How to achieve higher efficacy of T cell and B cell response, longer memory, broader protection, local mucosal protection, and minimal side effects remain important challenges in the field. Antigens and adjuvants are two classical components in vaccine design, and have been paid extensive attention and efforts in the past for vaccine development. It has been recognized that delivery systems endow the antigens and adjuvants with diversified functions. Specific delivery systems directly modulate distribution of both antigen and adjuvant spatially and temporally *in vivo*, therefore heavily influencing their biologic functions. A deeper understanding of how an antigen or an adjuvant works in the context of a specific delivery system *in vivo* will provide novel insights for the rational design of better vaccines.

This Research Topic aims to provide a forum to stimulate critical and creative thinking and showcase advanced research on the immunological mechanisms of vaccine delivery systems. Five representing original research articles have been published under this topic.

Beukema et al., using a whole inactivated influenza vaccine, investigated whether prolonged delivery of the influenza vaccine may improve the quality of the induced immune responses over that induced by prime-boost immunization. They immunized the mice with 5 µg of whole inactivated influenza vaccine derived from H1N1/2009 influenza virus through daily subcutaneous injections for a period of 14 days, 21 days, or 28 days, or through intramuscular prime-boost immunization. The results show that the highest levels of cellular and humoral immune responses were induced by 28 days of extended antigen delivery, followed by 21, and 14 days of delivery, and prime-boost immunization. Moreover, prolonged vaccine delivery also improved the quality of the induced antibody response, as indicated by a higher level of high avidity antibodies, a balanced IgG subclass profile, and a higher level of cross-reactive antibodies. This study has important implications for the design and development of future slow-release influenza vaccines.

Maina et al. investigated mucosal immunization regimens using a bovine RSV (BRSV) vaccine. The vaccine is a polyanhydride-based nanoparticle encapsulating the BRSV post-fusion F and G glycoproteins and unmethylated cytosine–guanine dinucleotide (CpG) motif. The vaccine was delivered prime-boost via heterologous (intranasal/subcutaneous) or homologous (intranasal/intranasal) immunization in a calf model. A commercial modified-live BRSV vaccine was used as a positive control. The results showed that the heterologous nanovaccine regimen induced both virus-specific cellular immunity and mucosal IgA, and induced similar clinical, virological and pathological protection as the commercial modified-live vaccine. The polyanhydride-based nanoparticle provides a flexible and safer design allowing for tunable antigen release kinetics and diverse antigen loading strategies.

Hartmann et al. optimized a transcutaneous vaccination delivery strategy for enhanced anti-tumor cellular immune responses. Transcutaneous immunization (TCI) is a non-invasive vaccination method. The authors had previously developed a combined application of the Toll-like receptor 7 (TLR7) agonist imiquimod (IMQ) together with the anti-psoriatic drug dithranol as an innovative TCI platform DIVA (dithranol/IMQ based vaccination). In the current study, they further optimized DIVA in terms of drug dose, application pattern and established a new IMQ formulation. The optimized transcutaneous vaccination method leads to the generation of a strong cellular immune response enabling the effective control of tumor growth and has the potential for clinical development as a novel non-invasive vaccination method for peptide-based cancer vaccines in humans.

Kim et al. tested the efficacy of Dectin-1 agonist, β-D-glucan, as an immunomodulatory adjuvant in a foot-and-mouth disease (FMD) vaccine model. Current FMD vaccines use a combination of inactivated viral antigens and oil adjuvants (emulsions) to enhance their efficacy. However, most commercial FMD vaccines have several limitations, such as low antibody titers, short-lived effects, compromised host defense, and safety issues. In the current study, the authors assessed the performance of β-D-glucan as a novel FMD vaccine adjuvant and found that it modulates both innate and adaptive immune responses and simultaneously enhances cellular and humoral immune responses in the host animal models, specifically pigs. This study provides a promising approach to overcoming the limitations of conventional FMD vaccines, and may also be helpful for the development of other vaccines.

Gary et al. explored how to induce potent adaptive immune responses in the aged, a major unmet need. The authors used a full-length SARS-CoV-2 spike DNA plasmid antigen (Wuhan D614G variant; pS) as a vaccine model and examined the immune responses in young (6-8 week-old) and aged (68-72 week-old) mice. Significant impairment of cellular responses and Th2-skewed humoral responses to spike antigens were observed in aged mice compared to those found in their young counterparts. Surprisingly, codelivery of a plasmid-encoding adenosine deaminase-1 (pADA) enhanced both the magnitude and functional capacity of serum antibodies in aged mice. Molecular mechanisms were also investigated. In summary, the authors reported a novel genetic adjuvant pADA that can enhance immune responses in aged mouse models of immunization and challenge.

Vaccine delivery systems are highly diversified, and many new systems are emerging. Advances in nanotechnology are providing us with materials that will permit tunable delivery systems. However, their immunological mechanisms remain poorly understood. Systemic investigation and deciphering the underlying mechanisms of spatial and temporal vaccine delivery may shed light on vaccine development in future. We hope this Research Topic will call more attention to this issue.

## Author contributions

MZ: Writing – original draft, Writing – review & editing. AW: Writing – review & editing. WL: Writing – review & editing.
